# Supplementary material for: Structural basis of aggregate binding by the AAA+ disaggregase ClpG
Source: J Biol Chem. 2023 Oct 10;299(11):105336. doi: 10.1016/j.jbc.2023.105336 (PMC10641755; doi:10.1016/j.jbc.2023.105336)
Supplement: Supporting Table S1 and References [file mmc2.docx]

Table S1: strains and plasmids used in this study

| **Strain** | **Description** | **Source or reference** |
| --- | --- | --- |
| *E. coli* XL1 blue | *recA1 endA1 gyrA96 thi-1 hsdR1 supE44 relA1 lac* [F’ *proAB* *lacI^q^ ΔM15* Tn10 (Tcr)] | Stratagene |
| *E. coli* BL21 | *F- ompT lon hsdSB gal dcm λ* (DE3) | Novagen |
| *E. coli* *ΔclpB* | MC4100 Δ*clpB*::Km | (1) |
| *P. aeruiginosa SG17M ΔclpB ΔclpG ΔclpG_GI_* | *P. aeruginosa* SG17M derivative strain | (2) |
| **Plasmid** | **Description** | **Source or reference** |
| pET24a-*clpG_GI_* | Vector for IPTG-inducible expression of *clpG_GI_* in *E.coli* BL21 cells | (2) |
| pET24a*-∆N1-clpG_GI_* | Vector for IPTG-inducible expression of *∆N1-clpG_GI_* (∆1-106) in *E.coli* BL21 cells | (2) |
| pET24a*-∆2-46-clpG_GI_* | Vector for IPTG-inducible expression of *∆2-46-clpG_GI_* in *E.coli* BL21 cells | This study |
| pET24a*-∆47-71-clpG_GI_* | Vector for IPTG-inducible expression of *∆47-71-clpG_GI_* in *E.coli* BL21 cells | This study |
| pDS56-*clpB-K476C* | Vector for IPTG-inducible expression of *clpB-K476C* in *E.coli* *ΔclpB* cells | (4) |
| pET24a-*N1-clpB-K476C* | Vector for IPTG-inducible expression of *N1-clpB-K476C* *(∆N-clpB-K476C* (∆1-145) fused with the N1-domain of *clpG_G_*_I_ (1-46)) in *E.coli* BL21 cells | This study |
| pDS56-*∆N-clpB-K476C* (∆1-145) | Vector for IPTG-inducible expression of *∆N-clpB-K476C* (∆1-145) in *E.coli* *ΔclpB* cells | (4) |
| pDS56-*∆N-clpB-E218A-K476C-E618A* (∆1-145) | Vector for IPTG-inducible expression of *∆N-clpB-E-218A-K476C-E618A* (DWB) (∆1-145 in *E.coli* *ΔclpB* cells | this study |
| pET24a-*clpG_GI_ -1-82* | Vector for IPTG-inducible expression of *clpG_GI_ -1-82* in *E.coli* BL21 cells | This study |
| pET24a-*clpG_GI_ -1-46-Strep* | Vector for IPTG-inducible expression of *clpG_GI_ -1-46* harboring a C-terminal Twin-Strep-Tag in *E.coli* BL21 cells | This study |
| pET24a-*clpG_GI_ -1-46- C6A/H34A-Strep* | Vector for IPTG-inducible expression of *clpG_GI_ -1-46-C6A-H34A* harboring a C-terminal Twin-Strep-Tag in *E.coli* BL21 cells | This study |
| pET24a-*clpG_GI_ -1-46- V17E-Strep* | Vector for IPTG-inducible expression of *clpG_GI_ -1-46-V17E* harboring a C-terminal Twin-Strep-Tag in *E.coli* BL21 cells | This study |
| pET24a-*clpG_GI_ -1-46- L21E-Strep* | Vector for IPTG-inducible expression of *clpG_GI_ -1-46-L21E* harboring a C-terminal Twin-Strep-Tag in *E.coli* BL21 cells | This study |
| pET24a-*clpG_GI_ -1-46- V17E-L21E-Strep* | Vector for IPTG-inducible expression of *clpG_GI_ -1-46-V17E-L21E* harboring a C-terminal Twin-Strep-Tag in *E.coli* BL21 cells | This study |
| pET24a*-clpG_GI_* -V17E | Vector for IPTG-inducible expression of *clpG_GI_ -V17E* in *E.coli* BL21 cells | This study |
| pET24a*-clpG_GI_* -L21E | Vector for IPTG-inducible expression of *clpG_GI_ -L21E* in *E.coli* BL21 cells | This study |
| pET24a*-clpG_GI_* -V17E-L21E | Vector for IPTG-inducible expression of *clpG_GI_ -V17E-L21E* in *E.coli* BL21 cells | This study |
| pUHE21 | Vector for IPTG-inducible gene expression in *E.coli* | (3) |
| pUHE21-*clpG_GI_* | Vector for IPTG-inducible expression of *clpG_GI_* in *E.coli* *∆clpB* cells | (2) |
| pUHE21-*∆N1-clpG_GI_* (*∆*1-106) | Vector for IPTG-inducible expression of *N1-clpG_GI_* (*∆*1-106) in *E.coli* *∆clpB* cells | (5) |
| pUHE21-*clpG_GI_ -C6A* | Vector for IPTG-inducible expression of *clpG_GI_ -C6A* in *E.coli* *∆clpB* cells | This study |
| pUHE21-*clpG_GI_ -H34A* | Vector for IPTG-inducible expression of *clpG_GI_ -H34A* in *E.coli* *∆clpB* cells | This study |
| pUHE21-*clpG_GI_ -C6A/H34A* | Vector for IPTG-inducible expression of *clpG_GI_ -C6A/H34A* in *E.coli* *∆clpB* cells | This study |
| pDK66-*yfp* | Vector for IPTG-inducible expression of *yfp* in *E.coli* *∆clpB* cells | Bukau lab |
| pDK66-*clpG_GI_ -yfp* | Vector for IPTG-inducible expression of *clpG_GI_* tagged with *yfp* at its C-terminus in *E.coli* *∆clpB* cells | This study |
| pDK66-*clpG_GI_ -V17E-yfp* | Vector for IPTG-inducible expression of *clpG_GI_ -V17E* tagged with *yfp* at its C-terminus in *E.coli* *∆clpB* cells | This study |
| pDK66-*clpG_GI_ -L21E-yfp* | Vector for IPTG-inducible expression of *clpG_GI_ -L21E* tagged with *yfp* at its C-terminus in *E.coli* *∆clpB* cells | This study |
| pDK66-*clpG_GI_ -V17E-L21E-yfp* | Vector for IPTG-inducible expression of *clpG_GI_ -V17E-L21E* tagged with *yfp* at its C-terminus in *E.coli* *∆clpB* cells | This study |
| pDK66-*clpG_GI_ -Q37E-yfp* | Vector for IPTG-inducible expression of *clpG_GI_ -Q37E* tagged with *yfp* at its C-terminus in *E.coli* *∆clpB* cells | This study |
| pDK66-*clpG_GI_ -L38E-yfp* | Vector for IPTG-inducible expression of *clpG_GI_ -L38E* tagged with *yfp* at its C-terminus in *E.coli* *∆clpB* cells | This study |
| pDK66-*clpG_GI_ -Q41E-yfp* | Vector for IPTG-inducible expression of *clpG_GI_ -Q41E* tagged with *yfp* at its C-terminus in *E.coli* *∆clpB* cells | This study |
| pDK66-*clpG_GI_ -K43E-yfp* | Vector for IPTG-inducible expression of *clpG_GI_ -K43E* tagged with *yfp* at its C-terminus in *E.coli* *∆clpB* cells | This study |
| pDK66-*clpG_GI_ -C6A-H34A-yfp* | Vector for IPTG-inducible expression of *clpG_GI_ -C6A-H34A* (*∆*Zn) tagged with *yfp* at its C-terminus in *E.coli* *∆clpB* cells | This study |
| pDK66-*clpG_GI_ -V17E-yfp* | Vector for IPTG-inducible expression of *clpG_GI_ -V17E* tagged with *yfp* at its C-terminus in *E.coli* *∆clpB* cells | This study |
| pDK66-*∆N1-clpG_GI_* (*∆*1-106) | Vector for IPTG-inducible expression of *clpG_GI_ -∆N1-clpG_GI_* (*∆*1-106) tagged with *yfp* at its C-terminus in *E.coli* *∆clpB* cells | This study |
| pJN105 | A broad-host range vector with arabinose inducible araBAD promoter, pBR1 origin of replication; Gm^R^ | (6) |
| pJN105-*clpG_GI_* | Vector for L-arabinose-inducible expression of *clpG_GI_* in *P. aeruginosa* cells | This study |
| pJN105-*clpG_GI_-C6A* | Vector for L-arabinose-inducible expression of *clpG_GI_-C6A* in *P. aeruginosa* cells | This study |
| pJN105-*clpG_GI_-H34A* | Vector for L-arabinose-inducible expression of *clpG_GI_-H34A* in *P. aeruginosa* cells | This study |
| pJN105-*clpG_GI_-C6A/H34A* | Vector for L-arabinose-inducible expression of *clpG_GI_-C6A/H34A* in *P. aeruginosa* cells | This study |
| pJN105-*clpG_GI_-V17E* | Vector for L-arabinose-inducible expression of *clpG_GI_-V17E*  in *P. aeruginosa* cells | This study |
| pJN105-*clpG_GI_-L21E* | Vector for L-arabinose-inducible expression *clpG_GI_-L21E* in *P. aeruginosa* cells | This study |
| pJN105-*clpG_GI_-V17E/L21E* | Vector for L-arabinose-inducible expression *clpG_GI_-V17E/L21E* in *P. aeruginosa* cells | This study |

**References**

1. Mogk, A., Schlieker, C., Strub, C., Rist, W., Weibezahn, J., and Bukau, B. (2003) Roles of individual domains and conserved motifs of the AAA+ chaperone ClpB in oligomerization, ATP-hydrolysis and chaperone activity. *J Biol Chem* **278**, 15-24

2. Lee, C., Franke, K. B., Kamal, S. M., Kim, H., Lunsdorf, H., Jager, J., Nimtz, M., Trcek, J., Jansch, L., Bukau, B., Mogk, A., and Romling, U. (2018) Stand-alone ClpG disaggregase confers superior heat tolerance to bacteria. *Proc Natl Acad Sci U S A* **115**, E273-E282

3. Mayer, M. P., Schroder, H., Rudiger, S., Paal, K., Laufen, T., and Bukau, B. (2000) Multistep mechanism of substrate binding determines chaperone activity of Hsp70. *Nat Struct Biol* **7**, 586-593

4. Oguchi, Y., Kummer, E., Seyffer, F., Berynskyy, M., Anstett, B., Zahn, R., Wade, R. C., Mogk, A., and Bukau, B. (2012) A tightly regulated molecular toggle controls AAA+ disaggregase. *Nat Struct Mol Biol* **19**, 1338-1346

5. Katikaridis P, Römling U, Mogk A. Basic mechanism of the autonomous ClpG disaggregase. Journal of Biological Chemistry [Internet]. 2021 Jan 1 [cited 2021 Sep 21];296:100460.

6. Newman JR, Fuqua C (1000_, Broad-host-ragne expression vectors that carry the L-arabinose-indcuible *Escherichia coli* *araBAD* promoter and the *araC* regulatior. *Gene* **227**: 197-203
